# Supplementary figures and images for: Graft–Host Interaction and Its Effect on Wound Repair Using Mouse Models
Source: Int J Mol Sci. 2023 Nov 13;24(22):16277. doi: 10.3390/ijms242216277 (PMC10671506; doi:10.3390/ijms242216277)

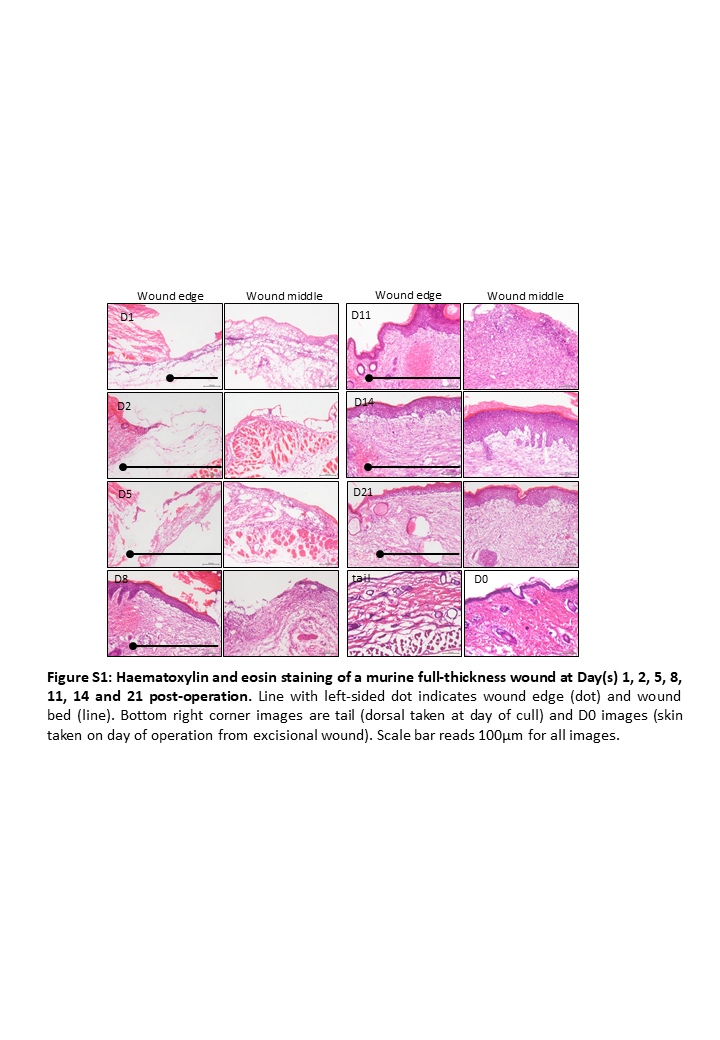

Supplement: Supplementary file 1 [file ijms-24-16277-s001.zip › Slide1.TIF]

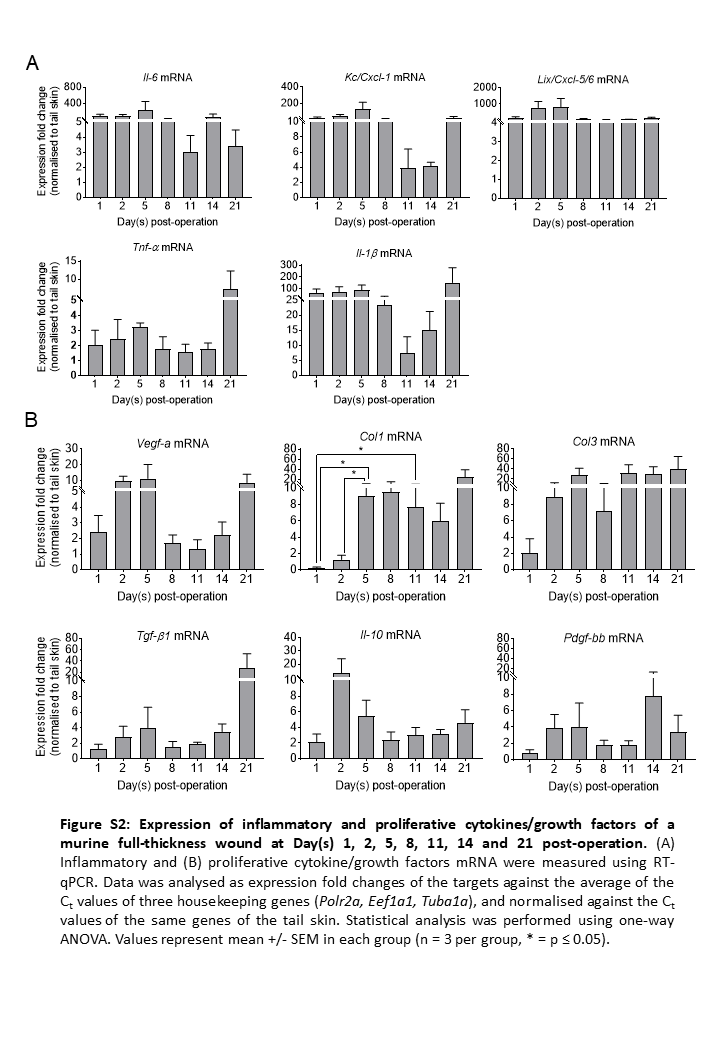

Supplement: Supplementary file 1 [file ijms-24-16277-s001.zip › Slide2.TIF]
